# Poly(ε-caprolactone) Nanoparticle Tumor-Lysate Vaccination in Mice Generates Hybridoma-Derived Antibodies Enabling Breast Cancer Diagnosis and Chemotherapy Synergy

**DOI:** 10.3390/biomedicines14010088

**Published:** 2026-01-01

**Authors:** Murat Ihlamur, Pelin Pelit Arayıcı, Emrah Şefik Abamor

**Affiliations:** 1 Department of Bioengineering, Faculty of Chemical and Metallurgical Engineering, Yildiz Technical University, 34220 Istanbul, Türkiye; pelinnpelit@gmail.com (P.P.A.); esabamor@gmail.com (E.Ş.A.); 2 Department of Electronics and Automation, Vocational School, Biruni University, 34015 Istanbul, Türkiye; 3 Health Biotechnology Joint Research and Application Center of Excellence, 34220 Istanbul, Türkiye

**Keywords:** poly(ε-caprolactone) tumor lysate vaccine, breast cancer, nanoparticle, hybridoma

## Abstract

**Background:** Tumor-lysate vaccines can capture tumor heterogeneity; however, their effectiveness may be reduced by antigen instability and short antigen presentation. Here, we aimed to improve antigen protection and prolong presentation by using a slow-degrading polymeric nanocarrier and an approved adjuvant. **Methods:** We encapsulated breast cancer cell lysates (MCF-7 and MDA-MB-231) in poly(ε-caprolactone) (PCL) nanoparticles using a double-emulsion (w/o/w) method and co-administered them with alum. We then characterized particle size, PDI, zeta potential, morphology, and in vitro release. Next, we evaluated nitric oxide (NO), TNF-α/IL-10 responses, and cytocompatibility in J774 macrophages. Finally, we quantified serum antibody titers in Balb/c mice after six biweekly immunizations, generated hybridomas, purified IgG, and tested antibody-mediated cytotoxicity alone and together with doxorubicin. **Results:** PCL nanoparticles were ~220–255 nm (PDI 0.10–0.19; ζ −2 to −3 mV) and released ~90–95% of encapsulated lysate by 800 h (~33 days). Encapsulated lysate (40 μg/mL) modestly increased NO versus control and increased further with alum (*p* < 0.05). TNF-α increased 7.4–9.72-fold, whereas IL-10 rose 2.82–3.11-fold. Importantly, encapsulated antigen + alum produced the highest ELISA responses after the sixth dose (6.36-fold for MCF-7 and 7.00-fold for MDA-MB-231 versus control; *p* < 0.05). Hybridoma-derived antibody signals increased through day 42, and Protein G purification yielded up to ~395 μg and ~318 μg IgG. Purified antibodies reduced cell viability, and viability decreased further when antibodies were combined with doxorubicin (to ~31.6% in MCF-7 and ~40.3% in MDA-MB-231). **Conclusions:** Overall, sustained PCL-mediated antigen release combined with alum strengthened humoral responses to tumor lysate and enabled recovery of functional antibodies with diagnostic capture and in vitro cytotoxic activity. In future work, key mechanistic steps such as lymph-node trafficking and cross-presentation should be tested directly.

## 1. Introduction

Breast cancer is among the most frequently diagnosed malignancies worldwide, and its contribution to morbidity and mortality is expected to continue rising in the coming years [1]. Data and projections from the WHO/IARC indicate substantial increases in both incident cases and cancer-related deaths [2]. This trend underscores the need—alongside primary therapies—for durable, antigen-specific vaccination strategies capable of establishing long-term immunity [3].

From an immunological standpoint, the success of therapeutic cancer vaccines hinges on efficient antigen uptake by antigen-presenting cells (APCs), timely access to draining lymph nodes, and—crucially—the ability to enter the MHC I pathway via cross-presentation [4]. Effective vaccination must not only initiate a robust primary effector response but also generate long-lived memory T and memory B cells [5]. Nanoparticle-based platforms developed in recent years can rationally tune many of these steps by facilitating intracellular targeting of antigen, enabling endosomal escape, and protecting cargo from proteolytic degradation [6]. Strategies that target dendritic cells (DCs) aim to exploit the superior cross-presenting capacity of human DC subsets (e.g., type 1 conventional dendritic cells (cDC1)) to amplify CD8^+^ T-cell responses [7]. Consequently, nanocarriers can reshape lymph node microarchitecture and antigen-presentation dynamics, and DC-targeted vaccination is increasingly viewed as translationally promising [8]. More broadly, nanomaterial-enabled platforms are being actively developed for tumor therapy and theranostics, including oxygenation strategies to modulate the tumor microenvironment and stimuli-responsive metal–complex systems for imaging-coupled drug delivery [9,10]. In parallel, rational nanocarrier design increasingly emphasizes transport barriers and structure–function relationships that govern tumor penetration and immune engagement [11,12,13].

Compared with vaccines focused on single epitopes, tumor-lysate–based approaches have the potential to better capture intratumoral heterogeneity through their multi-epitope content [14]. In breast cancer, DC vaccines loaded with lysate and other lysate-based formulations have been found safe in clinical/early studies and have shown signals of biological activity in terms of functional T-cell and humoral responses [15]. Nonetheless, these strategies often remain suboptimal due to challenges in antigen stability, lymph-node trafficking, and the need for sustained presentation [16]. Integration with carrier systems that protect lysate, enhance APC uptake, and provide controlled release can therefore markedly improve the quality of the immune response [17].

Biodegradable polymers are leading candidates for vaccine antigen delivery [16]. Within this class, poly(ε-caprolactone) (PCL) supports temporally extended antigen presentation owing to its slow, controllable degradation and is compatible with protein-friendly encapsulation methods such as double emulsion (w/o/w). PCL’s hydrophobic character can help preserve the structural integrity of protein antigens and shield them from proteolytic breakdown [18]. Experimental studies and reviews report that PCL nanoparticles can carry diverse protein/peptide cargos with low cytotoxicity, achieve prolonged release, and elicit both humoral and cellular immunity. In vaccination settings, PCL’s feasibility is further supported by release profiles consistent with the concept of single-dose, durable responses [19].

In comparison with PLGA-based systems, microenvironmental acidity is often discussed as a potential concern for protein stability; this context has motivated interest in polymers with comparatively neutral degradation profiles [20]. PLGA degradation products (lactic and glycolic acids) can acidify the particle interior, increasing the risk of structural damage to labile protein antigens; numerous formulations attempt to mitigate this with co-solvents, basic buffers, or stabilizers [21]. While studies detail how PLGA monomer ratios and surface modifications influence this microenvironment, PCL’s relatively neutral degradation profile can confer a stability benefit [22]. Given the premium on antigen integrity in cancer vaccines, these observations support PCL as an attractive nanocarrier [18].

Adjuvant choice is the second major axis shaping the phenotype and functional quality of vaccine responses [23]. Alum (aluminum salts), with a strong clinical track record, acts through multiple mechanisms, prominently including the NLRP3 inflammasome [24]. By amplifying innate immune signaling along the IL-1 family axis, alum enhances antigen uptake and cellular trafficking [25]. When these molecular effects are paired with a carrier that limits antigen loss and sustains presentation (e.g., PCL), there is an opportunity to jointly optimize both high-titer humoral responses and functional T-cell immunity [26]. Emerging work on nanoparticles and adjuvants also highlights NLRP3 as a central node in NP-mediated immunomodulation.

The effects of presenting breast cancer–derived multi-epitope lysate antigens via standardized encapsulation in a slow-release polymer such as PCL on key immunological endpoints—APC activation, cross-presentation efficiency, cytokine profiles, and memory dynamics—have been explored only in a limited number of studies [27]. Likewise, head-to-head comparisons that mechanistically delineate the response phenotype (Th1/Th2 balance, CD8^+^ cytotoxicity, long-term antibody maintenance) resulting from rational combinations of PCL-based platforms with alum—and that probe lymph-node targeting, inflammasome-dependent signaling, and endosomal escape—remain scarce [28]. While prior work separately suggests that lysate-based vaccines in breast cancer show biological activity and that PCL/NP carriers keep antigens stable and accessible, the integrated evaluation and systematic characterization of these two axes is still needed to drive translational progress [29].

**Figure 1:**
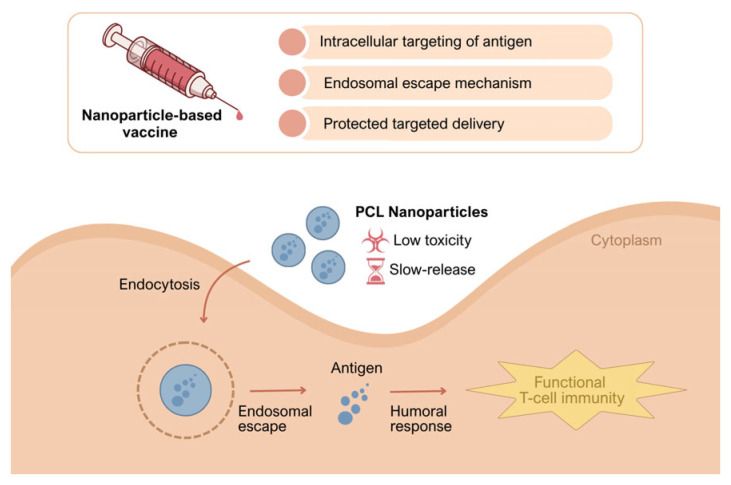
Conceptual mechanism of the PCL nanoparticle tumor-lysate vaccine. PCL nanoparticles are taken up by endocytosis, enabling endosomal escape and providing protected, slow release of tumor antigens, resulting in sustained antigen presentation and the induction of humoral responses together with functional T-cell immunity.

Addressing this gap, the present study investigates the packaging of breast cancer cell–derived multi-epitope lysate antigens within PCL nanoparticles to achieve sustained presentation, and examines the immunobiological performance that emerges when paired with alum [19]. We hypothesize that PCL-mediated controlled release will preserve antigen integrity, enhance APC uptake and cross-presentation, and thereby strengthen both primary and memory responses—optimizing high, durable antibody titers alongside functional CD8^+^ T-cell immunity [28]. We further posit that alum-driven innate signaling via NLRP3 will support these effects in the early phase, generating synergy that broadens and sustains the response [23]. In doing so, we aim to establish the immunological foundations of a broadly covering, clinically translatable vaccine architecture against heterogeneous breast tumors [27]. Accordingly, we designed a PCL nanoparticle–based tumor-lysate vaccine that combines protected delivery, intracellular targeting, and endosomal escape to promote durable antigen presentation (Figure 1). The schematic summarizes the hypothesized pathway by which this platform elicits both humoral responses and functional T-cell immunity.

## 2. Materials and Methods

### 2.1. Materials

High glucose dulbecco’s modified eagle medium (DMEM-H), Roswell Park Memorial Institute medium (RPMI 1640), fetal bovine serum (FBS), trypsin-EDTA, methyl thiazolyl tetrazolium (MTT), polyvinyl alcohol (PVA) (87–90% hydrolyzed, average MW 30.000–70.000 Da) were purchased from sigma-Aldrich (St. Louis, MO, USA). Penicillin, streptomycin, poly(ε-caprolactone) (PCL, average Mw ~14,000), and dichloromethane (DCM, hypergrade ≥ 99.9%) were obtained from Merck (Darmstadt, Germany). All cytokine kits were purchased from BTLAB (Bioassay Technology Laboratory, Shanghai, China).

### 2.2. Cell Cultures

The human breast cancer cell lines MCF-7 and MDA-MB-231 served as the experimental models in this study. Base media were enriched with 1% penicillin–streptomycin and 1% L-glutamine to prepare the culture conditions. For optimal growth, MCF-7 cells were propagated in DMEM supplemented with 10% FBS, while MDA-MB-231 cells were cultured in RPMI-1640 medium also containing 10% FBS. Cells used in experiments were between passages 10 and 15. All cell cultures were maintained under conventional incubation parameters of 37 °C, 5% CO_2_, and 95% humidity throughout the experiments. To enable large-scale antigen production by the sonication method for vaccine formulation, cell cultures were expanded in 75 cm^2^ polystyrene culture flasks (NEST, Wuxi, China). Once cell cultures reached approximately 80–90% confluence, enzymatic detachment was performed, followed by centrifugation at 1000 rpm for 5 min at 25 °C. The resulting pellet was rinsed with 1 mL phosphate-buffered saline (PBS) and subjected to a second centrifugation under colder conditions (1000 rpm, 5 min, 4 °C) to eliminate residual supernatant. The final pellet was then resuspended in 1 mL PBS and stored at −40 °C for subsequent applications [30].

### 2.3. Preparation of Breast Cancer Antigens by the Sonication Method

Breast cancer antigens were prepared from MCF-7 and MDA-MB-231 cell lines using the sonication technique. PBS-suspended cells, previously stored separately at −40 °C, were thawed in a 37 °C water bath, vortexed, and centrifuged at 1000 rpm for 5 min. After centrifugation, the supernatant was discarded, and the cell pellets were resuspended in 1 mL of PBS. The 15 mL Falcon tubes were attached to a sonicator probe and placed in an Erlenmeyer flask filled with ice-cold water and surrounded by ice. Sonication was performed for 40 s in 3 cycles, with the generator power set to 40%. This procedure was repeated 5 times. Following sonication, the tubes were centrifuged at 10,000 rpm for 3 min. The supernatant, containing the soluble antigenic components, was carefully collected [31]. The protein concentration in the lysates was determined using ultraviolet–visible (UV–Vis) spectroscopy at 280 nm and 260 nm wavelengths. Protein concentration was calculated using Equation (1). After quantification, the lysates were stored at −20 °C for subsequent experimental use.

Protein Amount = [(Abs 280 × 1.5) − (Abs 260 × 0.75)] × dilution factor (1)

Abs: absorbance value

### 2.4. Formulation of Nanoparticles (NPs)

Double emulsion solvent evaporation method (w/o/w) was used for encapsulation of MCF-7 cells antigen and MDA-MB-231 cells antigen into poly(ε-caprolactone) nanoparticles (PCL NPs). Briefly, 3% PCL was prepared in the organic solvent dichloromethane (DCM). MCF-7 cells antigen and MDA-MB-231 cells antigen were dissolved in milli-Q water. The dissolved antigens were added to PCL solutions in DCM and the mixtures for each formulation were then emulsified with sonicator (Bandelin Sonopuls) in an ice bath for 30 s (90 W amplitude). Afterwards the emulsion was poured by injector into 1%, *w*/*v* of an aqueous phase containing polyvinyl alcohol (PVA) as a stabilizer. The organic and aqueous phases were again emulsified with sonicator for 5 min 20 s. The obtained double emulsion (w/o/w) was stirred for 6 h at 1400 rpm to ensure complete evaporation of DCM. Furthermore, the obtained formulations of cell antigen–PCL nanoparticles were centrifuged at 14,000 rpm for 30 min and pellets were washed in three cycles with deionized water and the collected pellets were lyophilized for 30–48 h [32].

### 2.5. Characterization of Formulated Nanoparticles

Dynamic light scattering (DLS) (Zetasizer Nano ZS, Malvern, UK) was performed for determining the size, polydispersity index (PDI), and surface charge of each nanoformulation. Scanning electron microscopy (SEM) (ZEISS EVO 10, Oberkochen, Germany) was performed to elucidate the morphology of nanoparticles. To obtain release profiles of cell antigens, 10 mg of nanoparticles for each sample were dispersed in 5 mL PBS at pH 7.4 and then incubated in a shaking water bath at 37 °C. At different periods (0–800 h), sample solutions were centrifuged for 20 min at 12,000 rpm. The released amounts of antigens were determined by UV-Vis spectroscopy (JASCO V-530, Tokyo, Japan) at 280 nm, respectively. The encapsulation efficiency of NPs was determined by measuring the concentration of antigens obtained from the supernatant after ultracentrifugation of nanoparticles. The concentration of antigens was determined by comparing the concentration to an established standard calibration curve of cell antigens [33]. The encapsulation efficiency EE% was calculated based on the following equations:

EE (%) = (Amount of antigen determined in the nanoparticles (mg))/(Amount of initially added antigens (mg)) × 100(2)

### 2.6. Nitric Oxide (NO) Analysis

To assess the immunostimulatory potential of sonication-derived antigens, sonication-derived antigens-PCL and their combinations with adjuvants on macrophage cultures, NO production by the cells was measured using the Griess assay. Following a 24 h incubation of J774 macrophage cells at 37 °C in a 5% CO_2_ atmosphere, antigen solutions prepared at concentrations of 10, 20, 40, 80, 100 and 160 µg/mL were added to the cultures. After an additional 48 h incubation, the supernatants were collected and subjected to the Griess reaction. To prepare the Griess reagent, 1 g of sulfanilamide and 0.1 g of N-(1-naphthyl)ethylenediamine were dissolved in 2.5 mL of phosphoric acid, and the final volume was adjusted to 100 mL using distilled water. For nitric oxide quantification, 50 µL of culture supernatant from each well was dispensed into a 96-well microplate, followed by the addition of 50 µL of the freshly prepared reagent. The mixture was then incubated at room temperature for 10 min, after which absorbance was recorded at 540 nm using an ELISA-compatible microplate reader. After identifying the most effective antigen concentration based on NO levels, this optimal antigen dose was further evaluated in combination with alum and saponin adjuvants across the same concentration range (10–160 µg/mL), and NO production levels were again quantified [34].

### 2.7. Cell Viability Assay

J774 murine macrophage cells were maintained in T75 flasks using RPMI-1640 medium supplemented with 10% fetal bovine serum (FBS), 1% penicillin-streptomycin, and 1% L-glutamine. Cultures were incubated under standard conditions (37 °C, 5% CO_2_, and 95% relative humidity). When cell density reached approximately 80–90% confluence, cells were detached mechanically and collected by centrifugation at 1000 rpm for 5 min at 25 °C. Cell numbers were then determined using a Thoma hemocytometer (Isolab Laborgeräte GmbH, Wertheim, Germany). For the MTT-based cell viability assay, 1 × 10^4^ cells per well were seeded into 96-well culture plates and allowed to adhere for 24 h. Following this initial incubation, various vaccine formulations were introduced, and the cells were incubated for an additional 48 h. Cell viability was assessed using the MTT assay, employing 3-(4,5-dimethylthiazol-2-yl)-2,5-diphenyltetrazolium bromide. 10 µL of MTT solution was added to each well, and the plates were incubated in the dark at 37 °C for 3 h to promote the formation of formazan crystals. After incubation, the supernatants containing MTT were carefully removed, and 100 µL of dimethyl sulfoxide (DMSO) was added to each well to dissolve the formazan. The plates were then placed in the dark for 30 min before measuring absorbance at 570 nm using a microplate reader to quantify cell viability [35]. Each experimental condition was performed in triplicate, and mean values were calculated. Cell viability percentages were computed using Equation (1), and results were visualized through graphical representation.

Cell Viability (%) = (Sample absorbance/Control absorbance) × 100 (3)

### 2.8. In Vitro Quantification of Cytokine Levels

To evaluate the immunomodulatory effects of the developed vaccine formulations, the concentrations of IL-10 and TNF-α in the supernatants of J774 macrophage cultures were quantitatively determined using commercial enzyme-linked immunosorbent assay (ELISA) kits (BTLAB, Bioassay Technology Laboratory, Shanghai, China). Standard curves were established for each cytokine according to the manufacturer’s instructions using six concentration points: IL-10 (10–320 pg/mL) and TNF-α (40–1280 pg/mL). For each well, the total volume was adjusted to 100 µL. Specifically, 50 µL of either the standard solution or 40 µL of sample supernatant, along with 10 µL of biotin-labeled antibody solution, was added. Following this step, 50 µL of streptavidin–horseradish peroxidase (HRP) conjugate was added to each well, and the plates were incubated at 37 °C for 1 h. Upon completion of the incubation, wells were rinsed five times with a designated wash buffer to eliminate unbound reagents. Subsequently, 50 µL each of Substrate A and Substrate B was dispensed into the wells and incubated in the dark for 10 min to allow for color development. The enzymatic reaction was halted by introducing 50 µL of stop solution, and the optical density was read at 450 nm using a microplate spectrophotometer.

### 2.9. Experimental Animals

Animals were randomly assigned to treatment groups using a computer-generated sequence (block size = 5). All injections and sample processing followed this allocation. Outcome assessment (ELISA plate reading, NO/cytokine quantification) and the primary statistical analysis were performed blinded to group identity. Baseline blood sampling was completed prior to randomization, and group codes were concealed from analysts until all primary analyses were finalized. No animals were excluded unless pre-specified humane endpoints were met.

Vaccination and blood collection procedures were carried out at the Bezmialem Vakif University Experimental Animals Research Center after the approval of the Istanbul Bezmialem Vakif University Experimental Animals Ethics Committee (Ethical number: 2022/73). Balb/c mice were obtained from the Bezmialem Vakif University Experimental Animals Research Center, were maintained under specific pathogen–free (SPF) conditions, and had not undergone any prior experimental procedures before enrolment in this study.

All mice were housed in groups of five per individually ventilated cage in the Bezmialem Vakif University animal facility under controlled environmental conditions (temperature 22–24 °C, relative humidity 40–60%) with a 12 h light/12 h dark cycle. Standard rodent chow and tap water were provided ad libitum. Environmental enrichment, including nesting material and shelters, was available throughout the study.

Mice were monitored at least once daily for general health, grooming, posture, body condition, and behavior, and additionally on each vaccination and blood sampling day. Humane endpoints included >20% loss of initial body weight, marked lethargy, hunched posture, ruffled fur, impaired mobility, labored breathing, or inability to access food or water. Animals meeting any of these criteria were euthanized by CO_2_ inhalation followed by cervical dislocation. Intraperitoneal injections and tail vein blood sampling were brief procedures that, according to institutional guidelines, are associated with only mild and transient discomfort; therefore, no routine peri-procedural anesthesia or analgesia was used, but animals were observed closely after each manipulation. No unexpected adverse events were observed during the study.

The required sample size for the experimental animal studies was determined by statistical calculations, and the study was started with 5 female Balb/c mice (6 weeks old) in each group. Nine groups were created. Approximately 0.2 mL of blood was taken from each mouse for pre-vaccination baseline titer testing by tail vein sampling. The first group was designated as the control group. In this group, only the carrier solution (0.2 mL) was administered intraperitoneally. The second group was designated as the MDA-MB-231 Antigen group. In this group, 100 µg of sonicated MDA-MB-231 breast cancer antigen was administered intraperitoneally in a total volume of 0.2 mL. The third group was designated as the MDA-MB-231 Antigen + Adjuvant group. In this group, a mixture of 100 µg MDA-MB-231 antigen and 100 µg alum adjuvant was prepared and intraperitoneally administered with a volume of 0.2 mL. The fourth group was designated as the MDA-MB-231 Encapsulated Antigen group. In this group, 100 µg of encapsulated MDA-MB-231 antigen was administered intraperitoneally in a total volume of 0.2 mL. The fifth group was designated as the MDA-MB-231 Encapsulated Antigen + Adjuvant group. In this group, a mixture of 100 µg encapsulated MDA-MB-231 antigen and 100 µg alum adjuvant was prepared and intraperitoneally administered with a volume of 0.2 mL. The sixth group was designated as the MCF-7 Antigen group. In this group, 100 µg of sonicated MCF-7 breast cancer antigen was administered intraperitoneally in a total volume of 0.2 mL. The seventh group was designated as the MCF-7 Antigen + Adjuvant group. In this group, a mixture of 100 µg MCF-7 antigen and 100 µg alum adjuvant was prepared and intraperitoneally administered with a volume of 0.2 mL. The eighth group was designated as the MCF-7 Encapsulated Antigen group. In this group, 100 µg of encapsulated MCF-7 antigen was administered intraperitoneally in a total volume of 0.2 mL. The ninth group was designated as the MCF-7 Encapsulated Antigen + Adjuvant group. In this group, a mixture of 100 µg encapsulated MCF-7 antigen and 100 µg alum adjuvant was prepared and intraperitoneally administered with a volume of 0.2 mL [36].

Mice were vaccinated at two-week intervals until the antibody response was approximately 10 times higher than in the control group. Blood samples were collected before each vaccination.

### 2.10. Cell Fusion and Cloning

Peritoneal macrophages were prepared as a feeder layer prior to hybridoma generation. For this purpose, 10-week-old Balb/c mice from the control group were euthanized, and peritoneal cells were collected by lavage using RPMI-1640 medium. The immunized Balb/c mouse exhibiting an ELISA titer at least 10-fold higher than that of the control was then sacrificed, and splenocytes were aseptically isolated. Splenocytes were mixed with myeloma cells at a 10:1 ratio and fused using PEG 1500 (Sigma-Aldrich, St. Louis, MO, USA) in RPMI-1640 supplemented with 20% FBS. Following fusion, cells were centrifuged at 1250 rpm for 10 min and resuspended in RPMI-1640 containing 20% FBS and HAT supplement (hypoxanthine–aminopterin–thymidine). The cell suspension was distributed into 96-well plates and incubated at 37 °C in 5% CO_2_ for 14 days. On day 14, culture supernatants were collected to assess antibody production. Subsequently, HAT medium was replaced with HT medium (hypoxanthine–thymidine), and the cultures were maintained further. Antibody secretion was monitored by periodic sampling of hybridoma supernatants at defined intervals [37].

### 2.11. Enzyme-Linked Immunosorbent Assay (ELISA)

Breast cancer antigen obtained by the sonication method was diluted in 0.05 M carbonate coating buffer (pH 9.6) and used to coat 96-well microplates (100 µL/well; final antigen concentration 10 µg/mL). Plates were incubated overnight at 4 °C. The next day, wells were washed three times with PBS containing 0.05% Tween-20 (PBST). Non-specific binding sites were blocked with 2% (*w*/*v*) skimmed milk and incubated for 1 h at 37 °C. Serum samples were prepared at a 1:50 dilution in PBST supplemented with 2% milk, added to the wells, and incubated for 1 h at 37 °C. After washing, alkaline phosphatase-conjugated anti-IgG was diluted 1:1000 in PBST and dispensed into each well (100 µL/well), followed by incubation for 1 h at 37 °C. Wells were washed again, and p-nitrophenyl phosphate (pNPP; Sigma-Aldrich) substrate was prepared by dissolving 10 mg pNPP in 200 mL substrate buffer (0.02 g ZnCl_2_, 0.04 g MgCl_2_, and 1.5 g glycine; pH 10.4). Substrate solution was added to each well (100 µL/well) and the plate was kept for 30 min at room temperature in the dark. Absorbance was then read at 405 nm using an ELISA microplate reader (Thermo Fisher Scientific, Vantaa, Finland) [38].

### 2.12. Antibody Purification

IgG was purified from hybridoma culture supernatant on an ÄKTA purification system (UV280, conductivity, and pH monitoring enabled) (Cytiva Sweden AB, Uppsala, Sweden) using Protein G affinity chromatography. Cells were removed by centrifugation at 400–500× *g* for 10 min at 4 °C, and the clarified supernatant was passed through 0.45/0.22 µm filters and equilibrated in PBS. A HiTrap™ Protein G HP column (Cytiva, Uppsala, Sweden) was conditioned with dH_2_O and PBS, after which the filtered sample was loaded at 1 mL/min and the column was washed with PBS until the UV baseline stabilized. Elution was performed with 0.1 M glycine–HCl, and collected fractions were immediately neutralized in the collector tubes with 1 M Tris (pH 9.0; 5–10% of the fraction volume). Fractions corresponding to the UV280 peak were pooled, buffer-exchanged into PBS, sterile-filtered through 0.22 µm, and stored at 4 °C for the short term. Antibody concentration was determined by A280 (εIgG ≈ 1.4 [mg/mL]^−1^·cm^−1^) [39].

### 2.13. Antibody-Mediated Cytotoxicity ± Chemotherapy

MCF-7 and MDA-MB-231 cells were seeded at 1 × 10^4^ cells per well and incubated overnight (37 °C, 5% CO_2_). Antibodies were applied individually at doses of 0–20 µg/mL, and their cytotoxic effects on MCF-7 and MDA-MB-231 breast cancer cells were examined. Furthermore, combinations of the resulting antibodies were applied to doxorubicin and methotrexate at doses of 0–20 µg/mL. Plates were incubated for 48 h. Viability was then measured by MTT [35]. Each experimental condition was performed in triplicate, and mean values were calculated. Cell viability percentages were computed using Equation (3), and results were visualized through graphical representation.

### 2.14. Statistical Analysis

Animals were randomized to groups using a computer-generated sequence (block size = 5). Outcome assessment (ELISA plate reading, NO/cytokine quantification) and the primary statistical analysis were performed blinded to group identity. Group size (*n* = 5 per group) was chosen a priori from variability reported in comparable nanoparticle-vaccine studies (CV ~20–30%), assuming α = 0.05 (two-sided) and within-mouse correlation ρ ≈ 0.5 to provide ≥80% power to detect ~2-fold differences in ELISA titers; a ≤10% attrition margin was incorporated. For longitudinal ELISA readouts, per-time-point group comparisons were conducted by one-way ANOVA (Welch’s correction when variances were unequal), followed by Dunnett’s test versus the control at the matching time. Normality (Shapiro–Wilk) and variance homogeneity (Levene) were checked; data were log10-transformed when assumptions were violated. Results are reported as mean ± SD with exact *p*-values. As a summary measure, per-mouse area under the curve (AUC) across six biweekly doses was computed by the trapezoidal rule and compared by Welch ANOVA with Dunnett’s post hoc test. All ELISA analyses were performed in GraphPad Prism 9. For J774 macrophage studies, nitric oxide (NO) and cytokine (TNF-α, IL-10) levels were analyzed using one-way ANOVA followed by Tukey’s multiple-comparison test. In MTT assays, each condition was run in triplicate technical replicates; cell-viability percentages were calculated as (sample absorbance/control absorbance) × 100 and summarized graphically.

## 3. Results

### 3.1. Unencapsulated Antigen Analysis

#### 3.1.1. Determination of NO Activity

Nitric oxide (NO) is an important component of immunity and has two fundamental roles in the immune response: eliminating pathogens and regulating immune pathways. NO is a key molecule in the regulation of Th1 and Th2 responses. While low NO concentrations can stimulate Th2, high NO concentrations can support the Th1 immune response. In addition, Acosta et al. demonstrated a relationship between increased NO responses in vaccinated animals and the level of protection against the relevant pathogens [40]. Therefore, to evaluate the in vitro immunogenicity of the vaccine formulations, we investigated their effects on the concentrations of NO released by J774 macrophages. The level of NO released is an important tool to determine the potential stimulatory effects of vaccine formulations. In various studies, vaccine candidates have been shown to possess a strong ability to increase NO activity [41].

**Figure 2:**
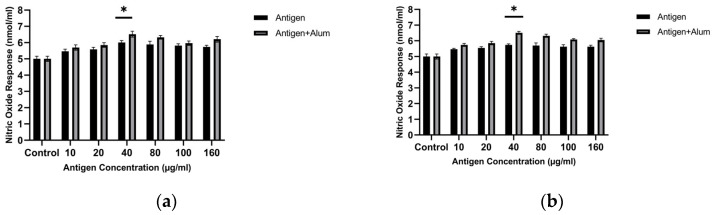
Nitric oxide (NO) production by J774 macrophages after exposure to unencapsulated breast cancer cell lysates with or without alum: (**a**) MCF-7 and (**b**) MDA-MB-231. NO was measured in culture supernatants using the Griess assay after incubation at the indicated lysate concentrations. * *p* < 0.05.

Initially, to determine the optimal antigen dose that best stimulates the NO response, increasing concentrations of sonicated antigens were applied to macrophages. The results show that the highest NO level was detected when macrophages were exposed to an antigen concentration of 40 µg/mL. Macrophage-derived nitric oxide (NO) was quantified to assess the immunomodulatory capacity of the antigen formulations. Treatment with 40 µg/mL MCF-7 whole-cell lysate resulted in a 1.2-fold increase in NO secretion compared with untreated controls, and treatment with 40 µg/mL MDA-MB-231 whole-cell lysate resulted in a 1.14-fold increase compared with untreated controls (*p* < 0.05, one-way ANOVA followed by Tukey’s test). This concentration was used in subsequent adjuvant combination studies (Figure 2). Based on these analyses, a concentration of 40 µg/mL for the sonicated antigens was selected for subsequent investigations.

Adjuvants are substances that enhance the immunogenicity of antigens, leading to stronger immune responses following vaccination [23]. In this study, we assessed the in vitro immunogenic properties of antigen–adjuvant combinations in terms of NO levels released from treated macrophages. The antigens were combined with alum, and the stimulatory activities of the combinations were compared with those of the antigens alone. The amounts of NO produced in the groups where macrophages were treated with antigen + adjuvant combinations were significantly higher than in macrophages exposed only to the pure antigens. In the study, the NO activity of the antigen + adjuvant combinations was higher than that of the antigens alone. Follow-up tests revealed that combining the antigens with adjuvants significantly increased NO secretion. The optimal concentrations were determined as 40 µg/mL antigen + 40 µg/mL alum for both cell lysates. The MCF-7 whole-cell lysate + alum induced a 1.3-fold increase, while the MDA-MB-231 whole-cell lysate + alum showed a 1.3-fold increase compared with the control (*p* < 0.05). These results indicate that both cell lysate antigens combined with adjuvant enhance the immunogenic profile (Figure 2).

#### 3.1.2. Cell Viability Analysis

**Figure 3:**
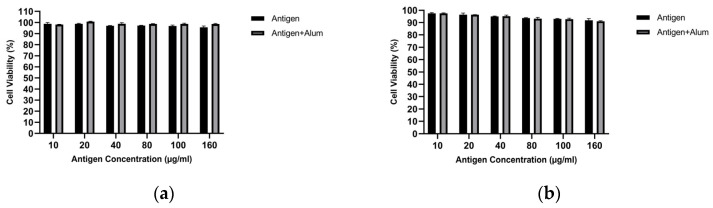
Cell viability of J774 macrophages measured by the MTT assay after treatment with unencapsulated breast cancer cell lysates in the absence or presence of alum: (**a**) MCF-7 and (**b**) MDA-MB-231 (concentrations as indicated).

MTT analysis was performed to determine the biocompatibility of the prepared vaccine formulations. The viability of treated macrophages was evaluated and compared for pure antigen versus antigen–adjuvant combinations. Figure 3 shows the cell viability of J774 macrophages following exposure to different vaccine formulations. As seen, at the tested concentrations, the antigens did not exhibit notable toxicity toward macrophages. In the antigen–adjuvant combinations, the 40 µg/mL antigen + 40 µg/mL adjuvant—which elicited the highest NO response—also provided an optimal toxicity profile. Therefore, subsequent immunization studies were designed with the vaccine formulation containing this mixture (40 µg/mL antigen + 40 µg/mL adjuvant), which is superior in stimulating in vitro NO responses and non-toxic to macrophages. All concentrations tested up to 160 µg/mL showed >85% viability, confirming that both the antigens and the antigen–adjuvant mixtures were non-toxic. The 40 µg/mL MCF-7 antigen + 40 µg/mL alum and 40 µg/mL MDA-MB-231 antigen + 40 µg/mL alum formulations showed >95% viability and were selected for animal immunization (Figure 3). These findings support the safety of the candidate vaccine formulations [42].

### 3.2. Encapsulated Antigen Analysis

#### 3.2.1. Zeta and SEM Analysis

**Figure 4:**
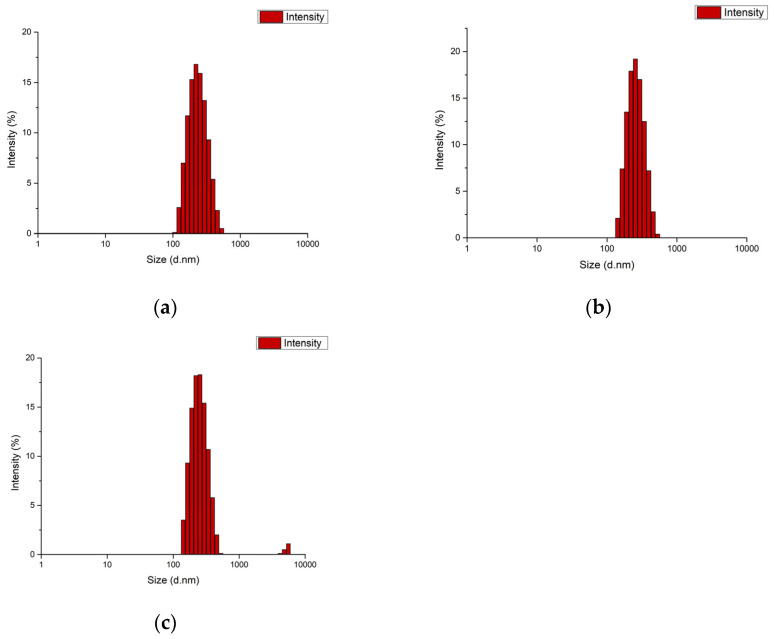
DLS size distribution of PCL nanoformulations: (**a**) blank PCL nanoparticles, (**b**) MCF-7 lysate–loaded PCL nanoparticles, and (**c**) MDA-MB-231 lysate–loaded PCL nanoparticles.

**Figure 5:**
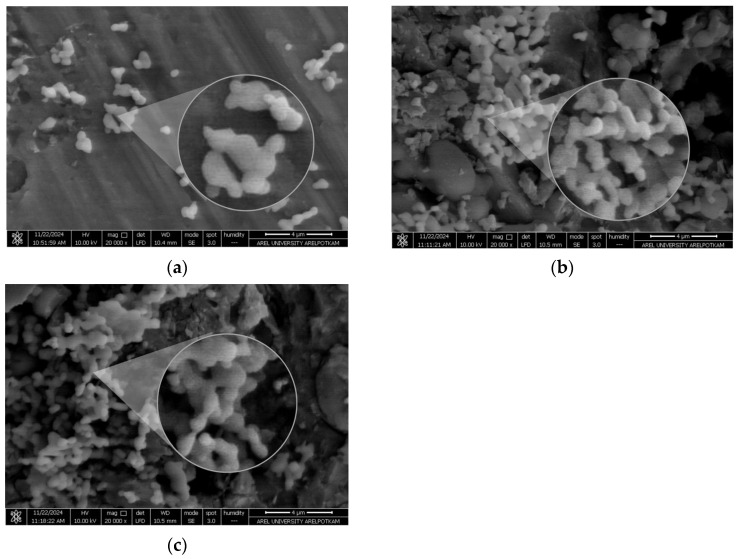
SEM micrographs of PCL nanoparticles showing particle morphology: (**a**) blank PCL nanoparticles, (**b**) MCF-7 lysate–loaded PCL nanoparticles, and (**c**) MDA-MB-231 lysate–loaded PCL nanoparticles. Scale bar: 4 µm.

The particle size of the blank PCL nanoparticle was determined as 220.5 nm, with a PDI of 0.098 and a zeta potential of −1.99 mV. The particle size of the MCF-7 antigen-loaded nanoparticle was 255 nm, with a PDI of 0.158 and a zeta potential of −2.75 mV. The particle size of the MDA-MB-231 antigen-loaded nanoparticle was 251.7 nm, with a PDI of 0.193 and a zeta potential of −3.22 mV (Figure 4 and Figure 5). It should be noted that apparent clustering observed in SEM micrographs may arise from sample drying during preparation; therefore, nanoparticle dispersion is more reliably assessed in conjunction with DLS size distribution and PDI measurements. The encapsulation efficiency (EE%) was 53.5% for MCF-7 lysate–loaded PCL nanoparticles and 56.8% for MDA-MB-231 lysate–loaded PCL nanoparticles.

#### 3.2.2. Release Analyses

**Figure 6:**
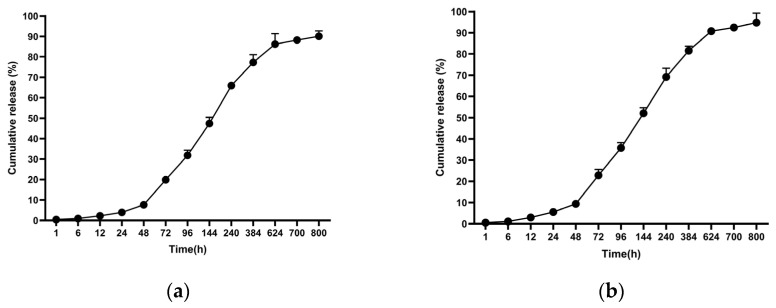
Cumulative antigen release profiles from PCL nanoparticles under physiological conditions (PBS, pH 7.4, 37 °C): (**a**) MCF-7 lysate–loaded nanoparticles and (**b**) MDA-MB-231 lysate–loaded nanoparticles. Data are presented as mean ± SD (n = 3).

In the release studies, PCL-encapsulated MCF-7 antigens released 90.07% at 800 h, whereas PCL-encapsulated MDA-MB-231 antigens released 94.78% at 800 h (Figure 6).

#### 3.2.3. Determination of NO Activity

To determine the optimal dose of encapsulated antigen that best stimulates the NO response, sonicated antigen concentrations were applied to macrophages. The results show that the highest NO level was detected when macrophages were exposed to 40 µg/mL of encapsulated antigen. Macrophage-derived nitric oxide (NO) was quantified to assess the immunomodulatory capacity of the antigen formulations. Treatment with 40 µg/mL encapsulated MCF-7 whole-cell lysate resulted in a 1.217-fold increase in NO secretion compared with untreated controls, while treatment with 40 µg/mL encapsulated MDA-MB-231 whole-cell lysate resulted in a 1.17-fold increase (*p* < 0.05, one-way ANOVA with Tukey’s test). This concentration was used in subsequent adjuvant combination studies. Based on these analyses, a concentration of 40 µg/mL for the encapsulated, sonicated antigens was selected for subsequent investigations.

**Figure 7:**
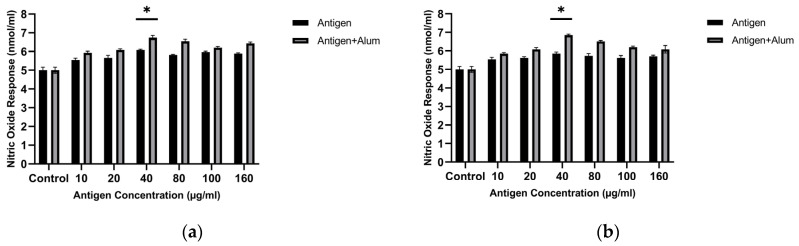
Nitric oxide (NO) production by J774 macrophages after treatment with PCL-encapsulated breast cancer lysates in the absence or presence of alum: (**a**) MCF-7 and (**b**) MDA-MB-231 (concentrations as indicated). * *p* < 0.05.

Encapsulated antigens were combined with alum, and the stimulatory activities of the combinations were compared with those of the encapsulated antigens alone. The amounts of NO produced in the groups where macrophages were treated with encapsulated antigen + adjuvant combinations were significantly higher than in macrophages exposed only to the encapsulated antigens. In this study, the NO activity of the encapsulated antigen + adjuvant combinations was higher than that of the encapsulated antigens alone. Follow-up tests revealed that combining the antigens with adjuvants significantly increased NO secretion. The optimal concentrations for both cell lysates were determined as 40 µg/mL encapsulated antigen + 40 µg/mL alum. The encapsulated MCF-7 whole-cell lysate + alum produced a 1.34-fold increase, while the encapsulated MDA-MB-231 whole-cell lysate + alum showed a 1.37-fold increase compared with the control (*p* < 0.05). These results indicate that, for both cell lysates, the encapsulated antigen + adjuvant combinations enhance the immunogenic profile (Figure 7).

#### 3.2.4. Cell Viability Analysis

**Figure 8:**
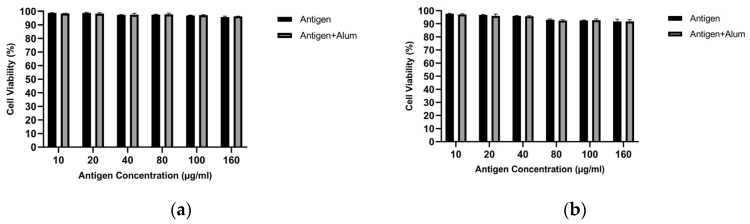
Cell viability of J774 macrophages measured by the MTT assay after treatment with PCL-encapsulated breast cancer lysates in the absence or presence of alum: (**a**) MCF-7 and (**b**) MDA-MB-231 (concentrations as indicated).

MTT analysis was performed to determine the biocompatibility of the prepared vaccine formulations. The viability of treated macrophages was evaluated and compared for encapsulated antigen versus encapsulated antigen–adjuvant combinations. Figure 8 shows the cell viability of J774 macrophages following exposure to different vaccine formulations. As observed, at the examined concentrations, the encapsulated antigens did not exhibit marked toxicity toward macrophages. In the encapsulated antigen–adjuvant combinations, the 40 µg/mL encapsulated antigen + 40 µg/mL adjuvant—which elicited the highest NO response—also provided an optimal toxicity profile. Therefore, subsequent immunization studies were designed with the vaccine formulation containing this mixture (40 µg/mL encapsulated antigen + 40 µg/mL adjuvant), which is superior in stimulating in vitro NO responses and non-toxic to macrophages. All concentrations tested up to 160 µg/mL showed >85% viability, confirming that both the encapsulated antigens and the encapsulated antigen–adjuvant mixtures were non-toxic. Accordingly, an IC50 value could not be calculated for the PCL-encapsulated antigen formulations (± alum) because 50% loss of viability was not reached within the tested concentration range. The 40 µg/mL encapsulated MCF-7 antigen + 40 µg/mL alum and 40 µg/mL encapsulated MDA-MB-231 antigen + 40 µg/mL alum formulations showed >95% viability and were selected for animal immunization (Figure 8). These findings support the safety of the candidate vaccine formulations [42].

### 3.3. In Vitro Measurement of Cytokine Production

**Figure 9:**
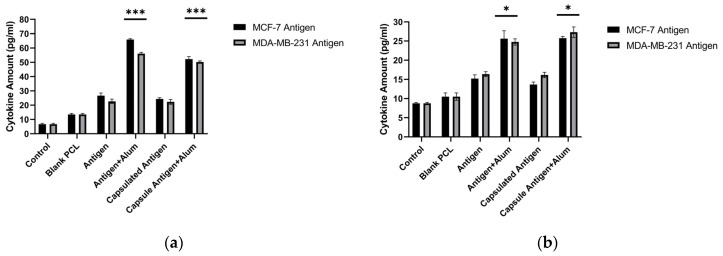
TNF-α (**a**) and IL-10 (**b**) production by J774 macrophages following treatment with free or PCL-encapsulated breast cancer lysates in the absence or presence of alum (ELISA). *** *p* < 0.001, * *p* < 0.05.

In response to the prepared formulations, TNF-α and IL-10 cytokine levels produced by J774 murine macrophages were quantitatively determined using commercial ELISA kits. Significant increases in cytokine levels were observed in the groups treated with antigen alone, encapsulated antigen, antigen + alum, and encapsulated antigen + alum. Since the goal of vaccine development is to strengthen the immune response by increasing antibody production, higher TNF-α levels together with relatively lower increases in IL-10 are considered desirable. Results for TNF-α and IL-10 are presented in Figure 9. In the formulations evaluated here, the concentrations of MDA-MB-231 and MCF-7 antigens and alum were applied at 40 µg/mL. Among the tested formulations, MCF-7 antigen + alum showed a 9.72-fold increase in TNF-α and a 2.92-fold increase in IL-10 relative to the control. Encapsulated MCF-7 antigen + alum showed a 7.69-fold increase in TNF-α and a 2.93-fold increase in IL-10 relative to the control. MDA-MB-231 antigen + alum showed an 8.26-fold increase in TNF-α and a 2.82-fold increase in IL-10 relative to the control. Encapsulated MDA-MB-231 antigen + alum showed a 7.4-fold increase in TNF-α and a 3.11-fold increase in IL-10 relative to the control (Figure 9) (*p* < 0.05).

### 3.4. Evaluation of Post-Immunization Antibody Titers

**Figure 10:**
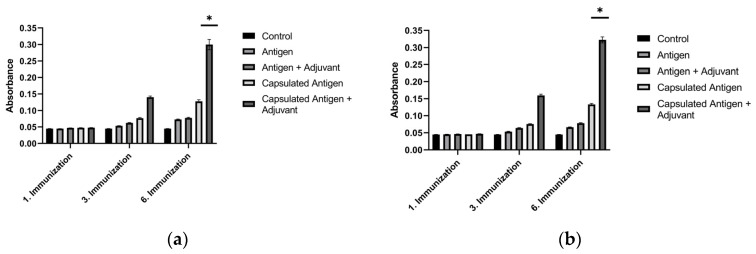
Serum antibody titers in Balb/c mice measured by ELISA over serial immunizations with breast cancer lysate formulations: (**a**) MCF-7 antigen and (**b**) MDA-MB-231 antigen. * *p* < 0.05.

To determine the in vivo immunostimulatory performance of the selected formulations, Balb/c mice were immunized with antigen–adjuvant and encapsulated antigen–adjuvant mixtures at the concentrations specified above. Compared with the control group, mice immunized with sonicated breast cancer antigens, encapsulated sonicated breast cancer antigens, and encapsulated sonicated breast cancer antigens combined with alum showed a clear increase. The most pronounced increase, however, was recorded in the group immunized with the combination containing encapsulated sonicated antigens and alum adjuvant. As clearly shown in Figure 10, baseline ELISA values in sera from the control groups ranged between 0.04 and 0.05. After the third immunization, antibody levels began to rise in the combination containing encapsulated antigen + adjuvant. This timing is consistent with a cumulative prime–boost effect, in which repeated antigen exposure supports germinal-center maturation, affinity selection, and expansion of antigen-specific plasma cells. Therefore, differences between formulations may become most apparent after multiple boosting rounds, once durable antibody-secreting populations are established. Following the sixth immunization with encapsulated MCF-7 antigen + alum adjuvant and encapsulated MDA-MB-231 antigen + alum adjuvant, the specific antibody levels against breast cancer antigens were 6.36-fold higher for the encapsulated MCF-7 antigen + alum adjuvant and 7-fold higher for the encapsulated MDA-MB-231 antigen + alum adjuvant compared with the control group (*p* < 0.05). The substantial increase detected in serum samples from these groups was considered sufficient for further hybridoma applications. These results indicate that encapsulated antigens are more potent than pure antigens in inducing immunogenic properties (Figure 10).

### 3.5. Antibody Levels Produced by Hybridoma Cells

**Figure 11:**
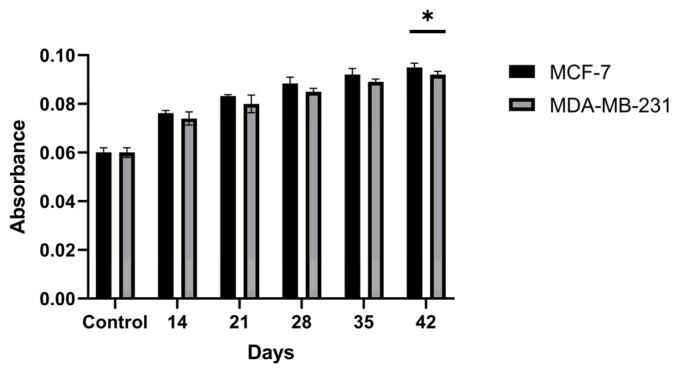
Time-dependent antibody secretion in hybridoma culture supernatants generated against PCL-encapsulated breast cancer lysates, quantified by ELISA at days 14, 21, 28, 35, and 42 post-fusion. * *p* < 0.05.

Antibody levels in hybridoma cells generated against the encapsulated breast cancer antigens were evaluated by ELISA on supernatants isolated from cultures on days 14, 21, 28, 35, and 42 post-fusion (Figure 11). The amounts of antibodies produced by the hybridoma cells were found to increase through day 42 of incubation. The antibody level of the hybridoma culture developed against the encapsulated MCF-7 antigen showed an ~1.58-fold increase on day 42 relative to the controls, while the hybridoma culture developed against the encapsulated MDA-MB-231 antigen showed an ~1.53-fold increase on day 42 relative to the controls (*p* < 0.05). These results indicate that the concentrations of antibodies secreted by the hybridomas increased markedly over the course of incubation.

### 3.6. Antibody Purification

**Figure 12:**
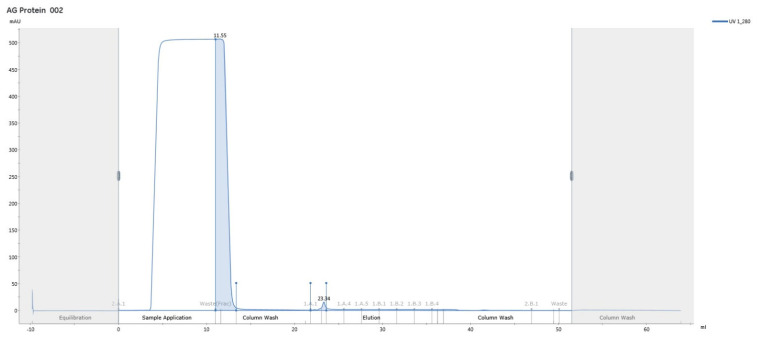
Representative ÄKTA Protein G affinity chromatogram (UV at 280 nm) showing purification of hybridoma-derived IgG from culture supernatant.

Antibody purification was performed on an ÄKTA system, and concentrations were determined by NanoDrop (version 2.0; Thermo Fisher Scientific, Wilmington, DE, USA). The yield of the MCF-7–derived antibody was 394.5 µg, and the yield of the MDA-MB-231–derived antibody was 318.2 µg (Figure 12).

### 3.7. Comparison and Diagnostic Value of the Produced and Commercial Antibodies

**Figure 13:**
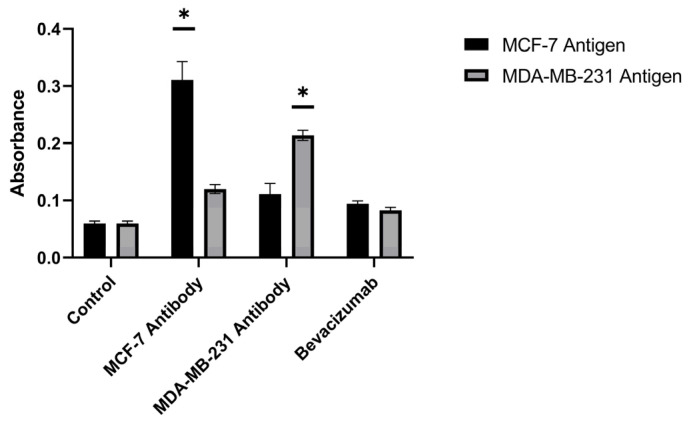
Comparative ELISA antigen-capture performance of purified hybridoma-derived antibodies (anti–MCF-7 and anti–MDA-MB-231) versus commercial bevacizumab against MCF-7 and MDA-MB-231 breast cancer antigens (cell lysates). * *p* < 0.05.

The diagnostic values of the antibodies produced against different breast cancer antigens and of commercially available antibodies were compared using the ELISA technique. The produced MCF-7 antibody exhibited a diagnostic value 5.18-fold higher than controls against MCF-7 breast cancer antigens and 2-fold higher than controls against MDA-MB-231 antigens. The produced MDA-MB-231 antibody exhibited a diagnostic value 1.85-fold higher than controls against MCF-7 breast cancer antigens and 3.56-fold higher than controls against MDA-MB-231 antigens (*p* < 0.05) (Figure 13). These outcomes indicate that the produced antibodies are effective in capturing antigens from both the MCF-7 and MDA-MB-231 cell lines.

### 3.8. Antibody Killing

**Figure 14:**
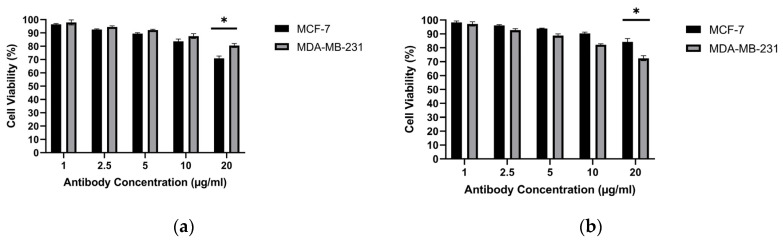
Cytotoxic effect of purified hybridoma-derived antibodies (IgG) on breast cancer cell viability: (**a**) MCF-7 and (**b**) MDA-MB-231, assessed by the MTT assay at the indicated antibody concentrations. * *p* < 0.05.

The cytotoxic activities of the obtained MCF-7 antibodies and MDA-MB-231 antibodies were examined against the MCF-7 and MDA-MB-231 cell lines. Antibodies produced from the MCF-7 cell line achieved 29.09% killing in the MCF-7 breast cancer cell line and 19.45% killing in the MDA-MB-231 breast cancer cell line. Antibodies produced from the MDA-MB-231 cell line achieved 15.74% killing in the MCF-7 breast cancer cell line and 17.51% killing in the MDA-MB-231 breast cancer cell line (Figure 14). These antibody-only cytotoxicity data form the basis for the subsequent combination analysis presented in Section 3.9.

### 3.9. Antibody–Drug Combination Cytotoxicity

**Figure 15:**
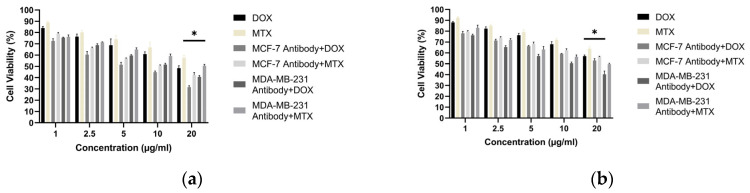
Cell viability of breast cancer cells treated with purified hybridoma-derived antibodies (IgG) in combination with doxorubicin: (**a**) MCF-7 and (**b**) MDA-MB-231, assessed by the MTT assay under the indicated treatment conditions. * *p* < 0.05.

Building on the antibody-only cytotoxicity results described in Section 3.7, we next evaluated antibody–doxorubicin combination effects. Antibodies generated by the hybridoma method from the MCF-7 and MDA-MB-231 cell lines were evaluated alone and in combination with doxorubicin (DOX) or methotrexate (MTX) for their cytotoxic effects on MCF-7 and MDA-MB-231 cells. Because the highest killing efficacy of antibodies derived from MCF-7 cells was observed at 20 µg/mL, this concentration was used for all antibody treatments. Drug combinations were tested at 1, 2.5, 5, 10, and 20 µg/mL. In MCF-7 cells, the greatest reduction in viability occurred with the MCF-7 antibody (20 µg/mL) combined with DOX, yielding 31.59% cell viability. In MDA-MB-231 cells, the highest killing efficacy was obtained with the MDA-MB-231 antibody (20 µg/mL) combined with DOX, yielding 40.27% cell viability (Figure 15).

## 4. Discussion

Encapsulating multi-epitope, breast cancer–derived lysate antigens in a protein-friendly polymer, poly(ε-caprolactone) (PCL), and pairing them rationally with alum establishes an integrated immune architecture that begins with early innate activation and extends through germinal center (GC) persistence to durable humoral immunity [43]. The physicochemical profile of our PCL nanoformulations—particle size ≤ 300 nm, narrow dispersity, and a mildly negative surface charge—is compatible with lymphatic transport in principle (Figure 4) [44]. Superimposed on this profile, the extended release kinetics (cumulative release > 90% by ~800 h, ≈33 days) maintain antigen availability over time (Figure 6), creating repeated, low-dose encounters with antigen-presenting cells (APCs), particularly dendritic cells [45]. Such a depot-like presentation prioritizes continuity over “sharp-peak/rapid-decay” exposure and widens the temporal window needed to establish and sustain GC reactions [43]. This presentation could facilitate antigen retention on the follicular dendritic cell (fDC) network and support iterative B-cell receptor selection; however, fDC-mediated retention remains to be established in dedicated studies [46]. In our study, PCL particles exhibited mean diameters of ~220–255 nm, PDI ~0.10–0.19, and zeta potentials of approximately −2 to −3 mV, while achieving cumulative release of ~90% (MCF-7) and ~95% (MDA-MB-231) by 800 h (≈33 days). Collectively, these data indicate that the formulation shapes immunity not only by delivering antigen, but also by tuning its presentation kinetics. In support of these physicochemical findings, SEM micrographs provided complementary visualization of the formulated particles, offering an orthogonal confirmation of the DLS-derived submicron size range and the absence of gross aggregation at the imaging scale (Figure 5). The agreement between DLS metrics and SEM observations confirms the intended submicron morphology and the absence of gross aggregation. While such features are consistent with efficient lymphatic transport, definitive confirmation will require biodistribution and LN-histology studies.

This spatiotemporal design is mirrored in the early innate phase by macrophage-derived nitric oxide (NO) and a pro-inflammatory cytokine signature. As an initial in vitro screen, unencapsulated lysate (± alum) increased macrophage NO at 40 μg/mL while maintaining high viability, supporting selection of this dose for subsequent studies (Figure 2 and Figure 3). Because NO serves as a quantitative barometer of the “danger” tone that supports APC activation and tissue-to-LN trafficking, its increase is mechanistically informative: At 40 μg/mL, encapsulated lysate elicited a moderate but significant increase in NO (MCF-7: ~1.22×; MDA-MB-231: ~1.17×; *p* < 0.05), which further increased with alum (MCF-7: ~1.34×; MDA-MB-231: ~1.37×; *p* < 0.05) (Figure 7). This pattern is consistent with alum’s NLRP3-centered activity operating alongside PCL-mediated sustained availability; it should therefore be interpreted as consistent with complementary effects rather than proven synergy. In parallel, a pronounced increase in TNF-α with comparatively limited increases in IL-10 suggests an overall pro-inflammatory balance within the tested conditions (Figure 9). This early signature could contribute to subsequent serological gains and sustained GC dynamics; putative upstream processes—such as T follicular helper (Tfh) differentiation, CD40L–BAFF engagement, and fDC-mediated antigen preservation—are proposed in the literature and will be clarified by dedicated analyses [47]. Importantly, these responses were achieved with preserved biocompatibility; selected combinations maintained >95% cell viability, a key prerequisite for translational development (Figure 8).

Within the adaptive arm of the response, the serological superiority of encapsulated-antigen + alum groups is particularly notable. Under a biweekly dosing schedule, antigen-specific antibody levels exceeded control after the sixth dose (MCF-7: ~6.36×; MDA-MB-231: ~7.00×), a pattern consistent with sustained humoral activation (Figure 10). Multi-epitope lysates strongly engage CD4^+^ T-cell help via MHC class II pathways and may also provide conditions permissive for cross-presentation to CD8^+^ T cells under appropriate particle–adjuvant contexts [48]. Functionally, the antibodies improved capture in ELISA-based assays (Figure 13) and reduced tumor-cell viability in vitro, consistent with Fc-dependent or direct effects that warrant further delineation (Figure 14) [49]. Combining the antibodies with doxorubicin further lowered viability in vitro (e.g., ~31.6% in MCF-7; ~40.3% in MDA-MB-231), suggesting an additional effect under co-treatment without invoking pharmacologic synergy (Figure 15). Together, these observations support the platform’s translational value in therapy-complementary settings (e.g., adjuvant/peri-adjuvant use, minimal residual disease windows). Consistent with these functional readouts, we were able to recover and quantify hybridoma-derived antibodies at the downstream processing stage. Hybridoma culture supernatants showed a time-dependent increase in antibody secretion over the monitoring period (Figure 11). Protein G–based purification on an ÄKTA system yielded 394.5 µg for the MCF-7–derived antibody and 318.2 µg for the MDA-MB-231–derived antibody (Figure 12) (NanoDrop A280), amounts sufficient for diagnostic capture assays, cytotoxicity testing, and early combination studies. These recoveries, obtained without extensive upstream optimization, indicate practical tractability of the platform and provide a baseline for scale-up (e.g., fed-batch hybridoma production or repeated column loading) in future translational work.

Synthesizing these findings, we propose the following working model. PCL’s slow, controllable erosion shields lysate antigens from proteolysis while supporting antigen availability and repeated encounters with APCs, in a manner consistent with retention on the fDC network and continued presentation on APCs [46]. Alum, through the NLRP3/IL-1 family axis, provides an early danger cue; the observed increases in NO/TNF-α and chemokine signals are consistent with enhanced innate activation, cellular trafficking toward LNs, and a milieu associated with Tfh support [24]. The multi-epitope cargo, in turn, supplies antigenic breadth that mitigates clonal narrowing around single epitopes and, when conditions permit, may engage cross-presentation pathways via specialized DC subsets (notably cDC1), potentially favoring CD8^+^ responses [50]. Acting together—continuity, spark, and breadth—these elements align with high-titer and functionally active antibody responses over the study period. Aligning the observed physicochemical profile and release kinetics with these immune outcomes within the same platform underscores a central principle: beyond dose, optimizing presentation kinetics can be an important determinant of immune quality.

PCL is favored here because its microenvironment better preserves protein integrity and its slow-release profile extends antigen availability [22]. While certain PLGA systems can develop an acidic internal microenvironment, PCL has been described with comparatively neutral degradation, a context compatible with maintaining lysate integrity [20]. In our experiments, PCL formulations exhibited prolonged release together with high antibody titers. Future work should include head-to-head comparisons between PCL and PLGA calibrated to the same cumulative release and initial size/zeta parameters, as well as rational combinations with alternative adjuvants (e.g., TLR agonists) to disentangle platform-specific contributions.

Despite these strengths, the present work is intentionally centered on humoral endpoints. Direct mapping of CD8^+^ T-cell cross-priming, Tfh/GC microarchitecture, and memory T-cell subsets should be pursued [51]. In parallel, in vivo LN biodistribution and verification of access to DC subsets (particularly cDC1) would strengthen the mechanistic link between particle size/surface chemistry and cellular trafficking. These considerations outline a roadmap for further testing of a ‘sustained-presentation plus early inflammatory spark’ model.

## 5. Conclusions

In this study, we formulated breast cancer lysates in PCL nanoparticles to protect the antigen and extend its availability over time. The resulting particles (~220–255 nm) provided a prolonged release window (~800 h), and alum further enhanced innate activation and antibody production. Consistent with this design, the encapsulated antigen + alum groups produced the highest serum titers and allowed us to generate hybridoma-derived IgG that improved ELISA-based antigen capture and reduced breast cancer cell viability in vitro. Moreover, combining antibodies with doxorubicin further decreased viability, suggesting an added benefit under co-treatment conditions. Taken together, these findings support PCL nanoparticles plus alum as a practical platform for generating diagnostically useful and functionally active antibodies from tumor lysates. Next, studies directly measuring antigen trafficking, cross-presentation, and efficacy in tumor-bearing models will be important to clarify the mechanism and translational relevance.

## Data Availability

The original contributions presented in this study are included in the article. Further inquiries can be directed to the corresponding author.

## Abbreviations

The following abbreviations are used in this manuscript:

Alum	Aluminum hydroxide adjuvant
DLS	Dynamic Light Scattering
DOX	Doxorubicin
ELISA	Enzyme-Linked Immunosorbent Assay
ER	Estrogen receptor
IgG	Immunoglobulin G
IL-10	Interleukin-10
NO	Nitric oxide
NP(s)	Nanoparticle(s)
PCL	Poly(ε-caprolactone)
PDI	Polydispersity index
SEM	Scanning Electron Microscopy
TNF-α	Tumor Necrosis Factor-alpha
w/o/w	Water-in-oil-in-water (double emulsion)
MTX	Methotrexate
PBS	Phosphate-buffered saline
